# Mast Cell-Mediated Mechanisms of Nociception

**DOI:** 10.3390/ijms161226151

**Published:** 2015-12-04

**Authors:** Anupam Aich, Lawrence B. Afrin, Kalpna Gupta

**Affiliations:** Vascular Biology Center, Division of Hematology, Oncology and Transplantation, Department of Medicine, University of Minnesota, Minneapolis, MN 55455, USA; aaich@umn.edu (A.A.); afrinl@umn.edu (L.B.A.)

**Keywords:** mast cells, pain, migraine, cytokines, cancer, inflammation, substance P, tryptase, hyperalgesia, sickle cell disease

## Abstract

Mast cells are tissue-resident immune cells that release immuno-modulators, chemo-attractants, vasoactive compounds, neuropeptides and growth factors in response to allergens and pathogens constituting a first line of host defense. The neuroimmune interface of immune cells modulating synaptic responses has been of increasing interest, and mast cells have been proposed as key players in orchestrating inflammation-associated pain pathobiology due to their proximity to both vasculature and nerve fibers. Molecular underpinnings of mast cell-mediated pain can be disease-specific. Understanding such mechanisms is critical for developing disease-specific targeted therapeutics to improve analgesic outcomes. We review molecular mechanisms that may contribute to nociception in a disease-specific manner.

## 1. Introduction

For nearly 150 years since the discovery of mast cells (MC) in 1863, first in frog tissue [1] and shortly thereafter in human tissue [2], the only MC disease known beyond allergic phenomena was the rare neoplastic mastocytosis in its various forms (cutaneous [3,4,5] and systemic [6,7]). Identification of MC products began in 1937 with the discovery that the metachromasia of MC granules is due to heparin [8,9], followed by finding of high MC histamine content in 1953 [10,11]. Through the remainder of the Twentieth Century there continued to evolve the modern understanding of not only the hematopoietic origin of the normally widely, sparsely distributed MC but also its fundamental function, namely, to produce and release a wide range of molecular signals, generally termed MC mediators, which contribute to the homeostasis of all cells, organs, tissues, and systems in the body. In 1987, tryptase was identified as a highly sensitive and specific marker for MC activation [12]. In time, though, the complexity of tryptase biology became more apparent [13], and it also became apparent that serum tryptase levels reflect the total body load of MCs far more than their activation state [14,15]. More than 200 MC mediators and cell-surface receptors are now known [16], underscoring the potential for heterogeneity of clinical MC-mediated disease pathologies.

Nociception is the process of transmitting sensation from the primary afferent neurons in the peripheral region to the brain via secondary neurons at the spinal cord level. Repetitive noxious stimulation of nociceptors contributes to central sensitization leading to chronic pain associated with number of diseases. Recently, interaction between immune and neural system, known as the neuroimmune interface, has been of immense interest for understanding the molecular mechanisms of peripheral and central sensitization underlying chronic pain [17]. Increased MC counts, enhanced MC degranulation, associated innervation, increased substance P (SP) and correlated hyperalgesia have been implicated in various chronic pain-associated pathologies [18]. The paracrine interaction between MCs and neural system at various levels of nervous system is complex, and the underlying molecular mechanisms are beginning to emerge. Contribution of mast cells to nociception appears to be critical in diverse pathological conditions, which has been highlighted in recently reviewed literature [18,19]. Hence, we provide a comprehensive review focusing on the molecular mechanisms of MC-mediated pain in disease-specific pathobiology and its clinical relevance.

## 2. What Are Mast Cells?

Mast cells (MCs) are tissue-resident granulocytes [20] that originate from CD34^+^/CD117^+^ myeloid progenitor cells in the bone marrow and circulate in blood during their immature stage. Subsequently stem cell growth factor (SCF) and other cytokines help the maturation of MCs in the vascularized tissue [21]. MCs reside in all tissues, predominantly those interacting with the external environment such as intestines, airways and skin [20], as well as in the dura mater at the spinal cord level and in the meninges of the brain [22]. Their proximity to the external environment enables MCs to be first responders to external pathogen and allergen exposure. Upon activation by external allergens or internal stimuli, MCs undergo degranulation and release pre-formed soluble mediators and those produced *de novo* upon stimulus [23]. Though traditionally MCs are known for mediating IgE-dependent allergic hypersensitive responses, they take part in tissue repair, cross-talk with other immune cells (such as T regulatory cells, B cells and Th17 cells), and recruit them at the site of injury or in case of pathogenic infection [20,24,25].

**Figure 1:**
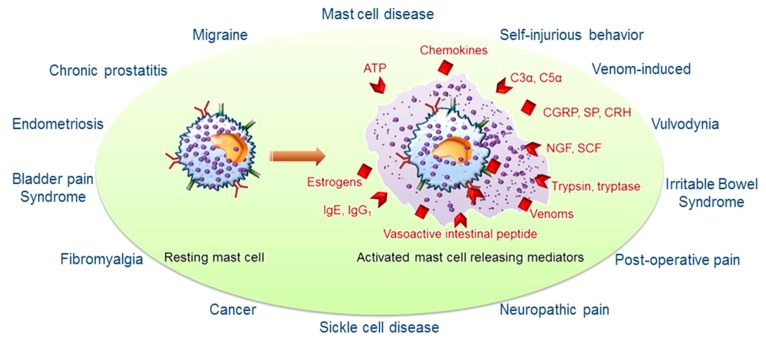
Mast cell-associated disease-specific pain syndromes, mast cell activation and its common activators: ATP (Adenosine tri-phosphate), chemokines, C3α, C5α (Complement 3α, 5α), estrogens, immunoglobins (IgE, IgG_1_), CGRP (calcitonin gene-related peptides), SP (substance P), CRH (corticotropin-releasing hormone), NGF (nerve growth factor), SCF (stem cell growth factor), trypsin, tryptase, venoms, vasoactive intestinal peptides.

Pre-formed MC mediators include proteases (e.g., tryptase, chymase, *etc.*), bio-organic amines (e.g., histamine and serotonin), proteoglycans (e.g., heparin, *etc.*), lysosomal enzymes, tumor necrosis factor alpha (TNFα) and others (e.g., nitric oxide synthase, endothelin and kinins) (see Figure 1). Newly synthesized mediators include lipid-derived prostaglandins and leukotrienes, cytokines (e.g., TNFα, MIF, interleukins and interferons), a large family of chemokines, growth factors (e.g., granulocyte macrophages colony stimulating factor (GM-CSF), nerve growth factor (NGF), stem cell factor (SCF), *etc.*) and antimicrobial species (e.g., antimicrobial peptides, superoxide, nitric oxide) [23]. (For a complete list of the mediators and triggers of MCs, see review [26]). MCs can also release neuropeptides such as substance P (SP), corticotropin-releasing factor (CRF), *etc.* [27]. The release of mediators depends on binding of specific stimuli to specific membrane-bound receptors, such as Fc family receptors, Toll-like receptors (TLRs), cytokine and chemokine receptors, neuropeptide receptors, complement receptors and hormone receptors [23]. Dysregulation of MC activation contributes to sustained inflammation and altered homeostatic imbalance via pro-inflammatory MC mediators which lead and/or contribute to diverse pathological conditions including, Alzheimer’s disease, anxiety, multiple sclerosis, rheumatoid arthritis, bladder pain syndrome, atherosclerosis, pulmonary hypertension, ischemia-reperfusion injury, irritable bowel syndrome, male infertility, obesity, diabetes mellitus and nociception [28].

## 3. Role of Mast Cells in Pain

MCs reside near the nerve fibers, which makes them an ideal candidate for modulating neural activity and nociception. MCs can interact with the nervous system bi-directionally, as MC-derived mediators such as tryptase and histamine lead to release of neuropeptides, e.g., SP and calcitonin gene-related peptide (CGRP) from the proximal nerve endings [29], and subsequently SP can further activate MCs [30]. Increased MC counts in proximity of neural system [31,32] and abnormalities in nerve fiber structure have been correlated with nerve growth factor (NGF), a MC-derived and nerve fiber-derived mediator which is a bidirectional source of hyperalgesia [33]. Enhanced MC degranulation and increased MC counts at the peripheral, dural and thalamic levels contribute to hyperalgesia in various rodent models of pain [34,35,36]. Considering the complex molecular origin of the neuroimmune interface between the nervous system and MCs, no universal model of molecular underpinning is sufficient to address such diverse pain behaviors. Therefore, we present a literature review focusing on disease-specific molecular mechanisms of MC-mediated pain pathobiology (for a summary, see Table A1 in the Appendix A).

### 3.1. Primary Mast Cell Disease-Related Pain

Primary MC disease, also known as MC activation disease (MCAD), encompasses two major disorders of MCs in the absence of any other diseases better fitting the entirety of the clinical picture. These two disorders are: (1) Mastocytosis—a rare condition of excessive MC proliferation in skin and circulation accompanied by release of MC mediators; and (2) MC activation syndrome (MCAS)—a more prevalent, relatively non-proliferative condition of enhanced MC activation [37,38]. Symptoms in MCAD are driven by increases in serum levels of MC-derived mediators and/or their urinary metabolites [38]. Serum tryptase usually is significantly elevated in mastocytosis but usually is normal in MCAS, in which other MC mediators typically must be explored to find evidence of MC activation. Symptoms of MC activation include hives; rash; anaphylaxis, joint, muscle and abdominal pain; hot flushing of skin, tachycardia; nausea; fatigue; and weight gain or loss. Pain in MCAD is common and is often described as migratory about a particular region of the body or even the entire body, with some patients complaining specifically of headache, epigastric pain, abdominal pain, skeletal pain, stomatitis, bone pain, muscle pain, pain in joints, ocular discomfort and ear/nose/throat inflammation [39,40]. In the largest symptom assessment in MC disease performed to date (420 patients, with 24% bearing cutaneous mastocytosis, 51% bearing systemic mastocytosis, and 12% bearing MCAS), pain was reported in the stomach, lower abdomen, joints, bones, muscle or nerve or connective tissue, upper abdomen, and chest by 73%, 56%, 61%, 56%, 50%, 46%, and 37%, respectively [41].

Given the complex networks of cascading effects resulting from MC mediator release, assigning a given pain sensation to a single etiologic molecular pathway would seem to be naively simplistic. The mutations in MC regulatory elements engendering aberrant MC mediator production and release [42,43] almost certainly originate in stem cells or pluripotent progenitor cells, implying the same mutations likely are present in other lineages, though the degree to which such mutations contribute to dysfunction in other lineages may be different than seen in MCs. Dysfunctional MCs may aberrantly release mediators constitutively and/or reactively. Furthermore, whether from MCs or other cells, aberrantly released mediators have potential to drive normal reactivity by other normal cells (MCs and otherwise) as well as normal and abnormal reactivity by other abnormal (*i.e.*, mutated) MCs and other cells. It also is the case that mediator effects can be localized or distant (via mediator escape into circulation or via engagement of local elements of the nervous and hormonal systems, which have their own transmission mechanisms). As such, multiple pathways leading to sensation of multiple types of pain can be quickly engaged, in acute or chronic fashion, and at sites local to, or distant from, a site of constitutive or reactive MC activation.

Treatment of pain in MCAD is often challenging, especially since many narcotic analgesics frequently are triggers and many patients also are triggered by the general class of non-steroidal anti-inflammatory drugs (NSAIDs). Even when adequately tolerated, narcotic and non-narcotic analgesics often are ineffective. Sometimes other classes of MC-targeted agents not expected to have analgesic effect are nonetheless strikingly effective at achieving such analgesic effects (e.g., antihistamines, leukotriene inhibitors, cromolyn, selective serotonin receptor antagonists) [44].

### 3.2. Migraine

Migraine is a neurological disorder characterized by recurring headaches of moderate to extreme severity associated with autonomic nervous system dysfunction, and it affects about 15% of general population [45]. More than five decades ago, Sicuteri suggested vasoactive and neuro-sensitizing roles for MCs in the complex pathology of migraine [46]. Years after this claim, increased histamine levels in migraine patients were observed [47,48,49] which could be antagonized by prophylactic anti-histamines [50,51,52] in a H1-receptor-specific manner in human subjects [53]. Other molecules usually associated with MC degranulation, such as endothelin-1 [54], TNFα and IL-6 [55], and leukotrienes [56], have been found to be associated with migraine in human subjects. Thus, MCs may contribute to migraine pain via several mechanisms.

The first direct evidence of degranulating dural MCs contributing to activation of trigeminal pain pathway via sensitization of meningeal and spinal-level trigeminal nociceptors was obtained in rats [35]. Further studies demonstrated that MC-derived mediators such as serotonin, prostaglandin, histamine (at a lesser extent), tryptase (via local protease-activated receptor 2 (PAR2) activation), TNFα (via cyclooxygenase (COX) receptors and the p38 mitogen-activated protein kinase (MAPK) pathway) and interleukin (IL)-1β, but not leukotriene, can sensitize meningeal nociceptors [57,58,59,60,61]. Contradictory effects of IL-6 on meningeal neuronal sensitization have also been reported [61,62].

Trigeminovascular stimulation in human and cats lead to increasing cranial blood flow which directly correlated with increased concentration of neuropeptides -SP and CGRP- in both trigeminal nerves and external jugular circulation [63]. Electric stimulation of trigeminal ganglion (simulating migraine-like pain) was associated with enhanced MC degranulation and subsequent plasma extravasation [64,65] in rats. This stimulated plasma extravasation and correlated MC activation in rat dura matter was blocked by treatment with serotonin agonists sumatriptan and dihydroergotamine, possibly via inhibiting neurotransmitter release [65]. A recent study has shown that extra- and intra-cerebral vaso-dilation is associated with migraine without aura, and sumatriptan (a serotonin agonist and well-known migraine medication) ameliorates migraine via vaso-constricting extra-cerebral arteries only [66]. From these results, it can be speculated that interplay of nerve fibers and MC degranulation via neuropeptides may provide both hyper-excitability of the meningeal neurons and vascular factors in the migraine pathology, while serotonin may promote anti-nociception at the dural level of the nervous system.

Acid-sensing ion channels (ASICs) are involved in sensitizing dural neurons in low pH conditions [67,68]. It is possible that MC degranulation can lead to lowering of local pH in the dura [69] and can sensitize the dural afferent nerves via ASICs [70]. Additionally, CGRP release from dural and trigeminal neurons is associated with lowering of pH [71]. Therefore, ASICs are important targets in MC-mediated migraine pathophysiology.

### 3.3. Pelvic Pain

Pelvic pain can originate from several disease conditions, some of which are gender-specific. We will be addressing three different chronic pelvic pain syndromes: (1) interstitial cystitis/bladder pain syndrome (gender non-specific); (2) chronic prostatitis associated chronic pelvic pain syndrome in men; and (3) chronic pelvic pain in women.

#### 3.3.1. Interstitial Cystitis/Bladder Pain Syndrome

Interstitial cystitis/bladder pain syndrome (IC/BPS) is a debilitating condition of pain and discomfort in the bladder of patients with urinary urgency and/or frequency which results in poor quality of life [72]. Among many implicated reasons, MC activation in IC/BPS has been studied widely [73]. Increased MC counts and infiltration in bladder wall along with increased levels of MC-released molecules (e.g., histamine, tryptase, IL-6, IL-8, NGF, *etc.*) in the urine of IC/BPS patients have been observed [74,75,76,77,78,79,80,81,82,83,84]. E-cadherin, a cell-cell adhesion molecule, has been found to be inversely correlated with increased MC infiltration, urothelial dysfunction and pain in IC/BPS patients [85,86]. MC-deficient mice showed reduced levels of experimental cystitis [87]. 

Various rodent models of cystitis [88,89,90,91] and a naturally occurring feline IC model [92] showed increased MC counts and activation. These models were associated with inflammation, tactile allodynia and hyperalgesia in the pelvic region. These studies revealed that MC involvement in neurogenic bladder inflammation and pain in cystitis [93] are in association with E-cadherin [94], via histamine and its receptors H1 and H2 on the bladder afferents [88,95], via both TNF and regulated on activation, normal T cell expressed and secreted (RANTES)-dependent [96,97] and -independent pathways [88,95], via neurokinin-1 (NK1) receptor [88], via chemokine (C-C motif) ligand 2 (CCL2)-mediated activation [98,99] and via Ca^2+^/calmodulin-dependent protein kinase II (CaMKII) [100]. It is possible that neurogenic sensitization of bladder nerve fibers lead to activation of MCs via SP action on NK1 receptors. Subsequent histamine release will further sensitize the afferents via histamine receptors and will result in sustained central sensitization via CaMKII induction [100].

#### 3.3.2. Chronic Prostatitis-Associated Chronic Pelvic Pain Syndrome

Chronic prostatitis-associated chronic pelvic pain syndrome (CP/CPPS) in the absence of bacterial infection is experienced by 15% of the male population and is characterized by urination urgency, burning sensation during urination and difficulty in urination [101]. CPPS patients (compared to controls) also showed increased MC counts and increased tryptase and NGF contents in the expressed prostatic secretion [102,103]. In the early 1980s in a rodent model of experimental auto-immune prostatitis (EAP), excessive MC degranulation was associated with CPPS in rats [104], while MC-deficient mice showed attenuated pelvic pain compared to EAP-induced controls [102]. These results demonstrate MC involvement in pain pathology of CP/CPPS.

Increased NGF availability may lead to increased MC recruitment leading to peripheral sensitization [105,106], and tryptase-PAR2-induced actuations of dorsal root ganglion (DRG) neurons may also be involved in sustained central sensitization [107]—both originating from MC-mediated neurogenic inflammation. MC-derived and MC-activating chemokines CCL2 and CCL3 may contribute to nociceptive sensitization at the spinal cord level [108]. Treating EAP with cromolyn (a MC stabilizer), histamine H1 receptor antagonist cetirizine and H2 receptor antagonist ranitidine—separately and in combination—resulted in effective reduction of chronic pelvic pain [102]. Therefore, histamine receptors on nerve cells may also be sensitized by histamine released by MCs. These results indicate involvement of MCs—via their mediators acting at multiple levels of the nervous system—in the pathogenesis of CP/CPPS.

#### 3.3.3. Chronic Pelvic Pain in Women

Chronic pelvic pain in women (CPPW) is characterized by loss of quality of life due to pain and discomfort in pelvic region lasting for more than six months and is not correlated with menstruation, sexual intercourse or pregnancy [109]. While the etiology of this pain in 61% of the 10 million affected women in the U.S. is unknown [110], CPPW is associated with endometriosis, IC/BPS, irritable bowel syndrome (IBS), musculoskeletal injury-induced pain, and nerve entrapment in scarred tissue [109]. Clinical endometriosis (EMS) is a painful estrogen-dependent disease of outgrowth of endometrium and is the major reason for CPPW. EMS has been shown to be associated with increased MC numbers and degranulation [111,112,113,114,115,116]. Increased levels of MC mediators such as IL-8, MCP-1, RANTES, and TNFα, and MC growth factor SCF have been found in the peritoneal fluid of human patients [117,118,119,120,121,122]. Toll-like receptor 4 (TLR4), which can activate MCs, is present extensively in EMS lesions [123]. Pain in deep infiltrating endometriosis has been found to correlate with increased MC infiltration and activated MCs in proximity to peripheral nerve endings, suggesting direct effect of MCs on peripheral sensitization [31]. In rodent models of endometriosis, increased MC infiltration is observed compared to control mice [124], and leukotriene antagonist treatment of rodent models reduced pain syndromes, indicative of MC-mediated CPPW [124,125]. Endometriotic lesions showed higher expression of SP, CGRP and NK1 receptors [126,127,128].

The mechanism of MC-mediated pain pathology in endometriosis is not clear. Allergen-induced IgE-mediated MC-activation may not be involved in EMS pain [129]; rather, a damage-associated molecular pattern (DAMP)-associated TLR-mediated MC activation model has been put forth [130]. The DAMP-TLR model of MC-mediated CPPW proposes that menstruation and EMS lesions can be source of DAMP molecules which activate MCs via TLR receptors. Upon activation, MC mediators can sensitize peripheral nerve endings, possibly via the transient receptor potential vanilloid subfamily 1 (TRPV1) channel [131]. Persistent sensitization will lead to release of SP and CGRP from nerve endings which can further increase MC activation, promoting sustained peripheral and central sensitization [130]. Moreover, prostaglandins can increase aromatase -induced estrogen biosynthesis, and estrogen can further increase expression of prostaglandins in EMS [132]. MCs can be activated by estrogen via the estrogen receptor and can release MC mediators including prostaglandins [133]. Therefore, multiple feed-forward loops of MC-activation (DAMP-TLR-mediated, neurogenic and estrogen-driven) may contribute to chronic pelvic pain in endometriosis.

### 3.4. Irritable Bowel Syndrome

Irritable bowel syndrome (IBS) is a functional disorder of the intestine characterized by bloating-gassiness of the bowel, diarrhea, constipation and abdominal cramping or pain. Increased MC counts in the jejunal, terminal ileal, cecal, colonic, and rectal mucosa of IBS patients compared to control individuals have been observed [134,135,136,137,138,139]. Increased tryptase levels were also found in colonic biopsies [137,138,139] and in the blood serum and intestinal fluid of IBS patients [134]. These results implicated a role of MCs in IBS pathobiology. Degranulating MCs located in increased numbers in the proximity (<5 µm) of colonic nerve fibers correlated with pain scores of the IBS patients [32,140,141] and were associated with increased SP and vasoactive intestinal peptide (VIP) [142]. Injecting supernatant from colonic biopsies of IBS patients increased visceral sensation in rats [143] and exhibited enhanced mesenteric afferent firing in a MC-dependent manner in rats and guinea pigs respectively [143,144]. Similar response was observed in human enteric neurons, while histamine receptor antagonists and protease inhibitors inhibited enhanced excitation [143,145]. In a small study of therapy-resistant IBS patients, evidence of pathologic MC activation was found in almost all the subjects [146]. MC stabilizers cromolyn and ketotifen reduced the visceral hypersensitivity in IBS patients [147,148]. These results suggest roles of MC infiltration and activation in IBS-related pain pathology. Also, increased intestinal permeability is correlated with increased nociception in IBS patients [141,149,150,151].

The nature of MC-mediated pain pathology in IBS patients is very complex and can result from multiple pathways. Proximity of MCs to nerve fibers may be of substantial importance for direct interaction between the two leading to increased sensation [32]. MC-derived tryptase may contribute to epithelial barrier dysfunction and hypersensitivity via PAR2 as evidenced from IBS patients and mice models of IBS [152,153]. PAR2 may sensitize neurons via Ca^2+^ mobilization [143,152] and TRPV1 channel activation [154]. MC-derived serotonin may contribute to pain in IBS as well [155,156]. Additionally, NGF contributes to visceral hyperalgesia in rats via modulating the plasticity of DRG neurons [157] and via mucosal dysfunction to promote MC-nerve direct interaction [158,159,160].

### 3.5. Vulvodynia

Vulvar pain that is chronic in nature with or without presence of sexual or other identifiable provocation is known as vulvodynia [161]. While no specific etiology has been established yet, vulvar nociceptor sensitization to mechanical and heat/cold stimuli have been recorded in human patients [162]. Increased MC infiltration and associated innervation in vulvodynia patients compared to controls have been reported in recent clinical histopathological studies of vestibular biopsies [163,164,165]. Chatterjea *et al.* have developed a female mouse model that exhibits MC-dependent sustained vulvar mechanical hyperalgesia and hyper-innervation upon topical administration of the hapten oxazolone [166]. Corroborating with high risk of vulvodynia in allergen-responsive individuals, Chatterjea *et al.* suggested allergic MC activation contributes to peripheral nociceptive sensitization in vulvodynia patients in absence of pruriception [19,167].

### 3.6. Complex Regional Pain Syndrome

Patients with confirmed and non-confirmed nerve injury from trauma to limbs (arms, legs, hands or feet) can suffer from disabling chronic pain, often referred to as complex regional pain syndrome (CRPS) [168]. Inflammatory cytokines such as TNF-α, array of interleukins, macrophage inflammatory protein (MIP)-1β and monocyte chemoattractant protein-1 (MCP-1) in blister fluid [169], IL-1β and IL-6 in cerebrospinal fluid [170], and neuropeptide SP [171] are suspected to be the primarysources of inflammation in CRPS [172]. MC-derived typtase levels in suction blister fluid showed a significant correlation with pain severity in clinically involved *vs.* uninvolved CRPS extremity [173], while increased MC accumulation is observed in skin biopsies of acute CRPS patients but not in chronic CRPS [174].

In a tibial fracture rat model of CRPS, inflammatory cytokines IL-1β, IL-8, TNFα and NGF have been shown to contribute to nociceptor sensitization [175,176,177,178,179,180]. In the same model, fracture-induced SP and CGRP release in sciatic nerve is seen with increased SP and CGRP gene expression in the ipsilateral DRG [181]. NK-1 receptor-dependent SP-mediated neurogenic vascular inflammation and pro-nociceptive pathology were observed in a CRPS rat model [171,177,181,182]. Finally, the same group demonstrated that SP signaling controls nociceptive sensitization due to MC-activation via NK-1 receptors—not histamine receptors—in rat CRPS [183]. Increased MC counts in the upper dermis and associated MC activation via SP in a CRPS rat model may also activate keratinocytes expressing histamine and PAR2 receptors, and subsequently PAR2 may activate proximal transient receptor potential cation channel subfamily A member 1 (TRPA1) leading to increased peripheral sensitization [183].

### 3.7. Venom-Induced Hyperalgesia

In experimental rat models of venom-induced hyperalgesia *Bothrops jararaca* snake venom [184], Batroxase metalloproteinase from *Bothrops atrox* snake venom [185] and *Buthus martensi* Karch (BmK) scorpion venom [186] induced nociceptive behaviors accompanied by MC degranulation and histamine release. This venom-induced hyperalgesia in rats was reduced by MC stabilizers cromolyn and sodium nedocromil [187], MC depleter compound 48/80 [186] and dexamethasone [185], and histamine receptor antagonists diphenhydramine, chlorpheniramine, pyrilamine and cimetidine [185,186].

Studies focusing on BmK venom have indicated that its peripheral administration contributes to nociception associated with increased c-Fos [188], neuropeptides, extracellular signal-regulated kinases (ERK), and nitric oxide activity in the spinal cord level [189]. While neurotoxins derived from BmK venom possibly contribute to peripheral neuronal hyper-excitation via site-specific action on voltage-gated sodium channels of DRG neurons [190,191], β/β-like neurotoxins can induce anti-nociceptive effects in rats via site-specific blocking effect on the voltage-gated sodium channels of the peripheral [192,193,194,195,196,197,198] and central neuronal cells [194,196,197,198,199]. It therefore is unclear which components of BmK venom may contribute to MC-mediated venom-induced hyperalgesia with involvement of complex signaling pathways for both peripheral and central sensitization [189].

### 3.8. Fibromyalgia

Fibromyalgia syndrome (FMS) is a neurobiological disorder characterized by pressure-induced pain in specific tender points in the muscles in all four quadrants of the body [200,201] and is associated with sleep disturbances, morning stiffness, fatigue, paresthesia, headache and anxiety—possibly induced by stress and other psychological factors [202]. The idea of MC-mediated peripheral and central sensitization in FMS originates from a line of studies showing increased MC infiltration [203] and increased MC degranulation with increased PAR2 activity [204] in skin biopsies, and increased levels of IL-1, IL-6, IL-8 and MCP-1 in the serum of FMS patients *vs.* healthy controls [205,206]. Though a recent phase 1 randomized clinical trial for treating FMS patients with the MC stabilizer ketotifen (2 mg twice daily) found no significant change in pain in FMS patients compared to placebo-treated patients, a higher dosage of ketotifen may be warranted [207].

A mechanism of MC-mediated nociception in FMS as a hypothesis for pain in FMS patients in addition to hypothalamic-pituitary-adrenal (HPA) axis involvement has been put forth [205]. The thesis speculates that MCs may be activated by local release of corticotropin-releasing hormone (CRH) and SP via CRH and NK1 receptors, respectively, and can lead to a feed-forward neuroendocrine sensitization of the peripheral and central nervous systems in FMS [208,209,210,211,212,213,214].

### 3.9. Self-Injurious Behavior-Associated Pain

Self-injurious behavior (SIB) is displayed by individuals with intellectual and related neurodevelopmental disorders [215]. SIB is characterized by a deliberate act of causing tissue-scarring damage to oneself in the absence of sexual arousal and conscious suicidal intention [216]. Subsets of adults and children (*vs.* non-SIB controls) with chronic self-injury demonstrated increased MC degranulation with increased innervation and SP in the skin biopsies [217,218,219,220]. Adults exhibited increased tactile sensation correlated with increased MC activity. In a rodent model cromolyn reduced stress-induced behavioral abnormalities [221]. Therefore, it is possible that stress-induced dysregulation of neuronal activity and repetitive injury in chronic SIB leads to release of neuronal SP due to hyper-excitation. This subsequently activates nearby MCs. In turn, activated MCs can lead to sustained peripheral sensitization via its mediators and via direct interaction with proximal increased dense nerve fibers.

### 3.10. Cancer-Associated Pain

Pain affects almost two-third cancer patient population, and about 50% patients demonstrate moderate-severe pain with poor quality of life and shorter life span in many cancer types [222]. Cancer progression and pain may be correlated via tumor innervation [223], substance P [224] or immunomodulatory endogenous mu opioid receptors [225]. Opioids are the mainstay for treatment of pain in cancer. In a retrospective study of metastatic advanced prostate cancer, we found that shorter life span was associated with higher opioid requirement; and that higher opioiod requirement was associated with pain and reduced survival in advanced non-small cell lung cancer patients [226,227]. MCs have been shown to be involved in tumor progression, innervation, associated pain conditions and shorter life span of patients [228,229,230] in pancreatic cancer. It is conceivable that morphine may act via activation of mast cells in the tumor.

We recently showed that morphine contributes to tumor progression (not the onset) via mu opioid receptor and MC activation in a murine breast cancer model, leading to shorter survival [231]. We observed increased immunoreactive SP co-localized with MCs and elsewhere in tumor in response to morphine treatment, with simultaneous increase in GM-CSF and RANTES. So it is possible that morphine-induced MC degranulation leads to SP release via tryptase-PAR2 action on peripheral nerves, which subsequently contributes to sustained neuro-inflammation and pain in cancer, consequently promoting increased opioid requirement and subsequent shorter life span. Thus, peripherally acting mu opioid-receptor and MC-targeted therapeutics may be beneficial in treating cancer pain without an inadvertent effect on cancer progression and survival.

### 3.11. Sickle Cell Pain

Sickle cell disease (SCD) was the first molecular disease to be identified due to a single nucleotide polymorphism of hemoglobin [232]. Under low oxygen, sickle hemoglobin polymerizes, leading to red blood cell (RBC) deformation into a sickle shape. These poorly deformable sickle RBCs block capillaries, leading to vaso-occlusive crises (VOC), which impair oxygen and nutrient supply to the tissues, and leading to inflammation, ischemia/reperfusion injury, oxidative stress, end-organ damage and severe pain. In addition to acute pain due to VOC, SCD can be accompanied by severe chronic pain which can start in infancy, leading to hospitalization, reduced survival and poor quality of life [233]. Only one case report of high concentration of MC tryptase in sickle patient’s blood is available; this patient with sickle cell/β-thalassemia and acute chest syndrome died due to overdose of fentanyl [234]. The MC inhibitor imatinib significantly attenuated pain crises in SCD patients in two case reports [235,236]. Treatment of co-morbid MCAS helped some SCD patients in a recently reported case series [237].

SP and CGRP released by sensitization of peripheral nociceptors lead to inflammation, vasodilation and plasma extravasation—which is referred to as neurogenic inflammation. We found that MC activation contributes to neurogenic inflammation in a transgenic mouse model of SCD [34]. We observed that MC activation is increased in sickle mice skin biopsies compared to control mice as evident by increased co-localization of tryptase with FcεRI and CD117. Expression of TLR4, is more than threefold higher in sickle mice skin *vs.* control mice. SP induced tryptase release from sickle MCs in culture, but not from control MCs, while SP and tryptase were elevated in tissue sections from sickle mice *vs.* control. Morphine induced increased release of SP and tryptase from MCs. Cromolyn and imatinib reduced SP, CGRP, tryptase, β-hexosaminidase and serum amyloid protein (SAP) in plasma of sickle mice after a five-day treatment, indicative of MC-mediated inflammation in sickle pathology. Cromolyn and imatinib treatment reduced, while morphine treatment induced, SP and CGRP release in skin and DRG compared to vehicle-treated sickle mice. This is indicative of pro-neuroinflammatory properties of morphine. Intradermal injection of capsaicin (a TRPV1 agonist that activates primary afferent neurons) or SP increased Evans blue (EB) leakage in sickle mice (*vs.* control mice), demonstrating existence of neurogenic inflammation in SCD. Cromolyn or imatinib treatment of sickle mice led to a significant decrease in EB leakage, confirming that MCs contribute to neurogenic inflammation in SCD, while morphine increased EB leakage in vehicle-treated sickle mice compared to control mice. Imatinib treatment of mice reduced inflammatory cytokines from skin biopsies *ex vivo*, abrogated tonic hyperalgesia and white blood cell count, and prevented hypoxia/reoxygenation-induced hyperalgesia in sickle mice. Sickle mice lacking MCs showed reduced level of hyperalgesia compared to sickle mice, demonstrating that mast cells contribute to hyperalgesia in sickle mice. Notably, cromolyn pretreatment of sickle mice improved analgesic effect of a sub-optimal dose of morphine in sickle mice morphine. It is likely that morphine acts as an analgesic via its MOR-mediated effect on the CNS, but activates mast cells, which contribute to increased nociception, thus reducing the effectiveness of morphine, leading to the requirement of a relatively higher dose of morphine to treat sickle pain.

Our findings detailed above show that MC-mediated neurogenic inflammation and hyperalgesia are involved in complex sickle pathobiology. We propose that neuropeptides (e.g., SP and CGRP) are released from peripheral nerve endings by action of MC-derived tryptase (via PAR2-TRPV1 pathway). SP contributes to further activation of MCs, leading to sustained release of SP and tryptase from MCs. Additionally, cytokines released from degranulation of activated MCs may also contribute to the inflammation and sensitization of peripheral and central nociceptors in SCD. Though we observed higher expression of FcεRI and CD117 in the biopsies (sickle *vs.* control), a non-pathogenic DAMP-associated activation of TLR4 may be the mediator of MC activation. Thus MCs play a significant role in a feed-forward cycle of neurogenic inflammation and pain in SCD pathobiology [34].

Inhibiting MC activation by MC stabilizers and/or inhibitors potentiates low-dose morphine’s analgesic action, and such combination therapy provides a direction for future clinical application to reduce requirement of high-dose analgesics. Tolerance is an important criterion when treating patients with analgesics. In a recent study we showed that a high affinity nociceptin- and mu-opioid receptor agonist, AT-200, induced analgesia in sickle mice with significant reduction of thermal, mechanical and deep-tissue hyperalgia in case of both chronic and hypoxia-reoxygenation-evoked acute pain [238]. AT-200 has low efficacy at the mu opioid receptor. Our results indicated that analgesic effects of AT-200 are mediated by its ability to reduce inflammation and MC activation without causing tolerance. Our recent data (unpublished) also show that cannabinoid receptors (CBR) activation holds promise for improved therapeutics, without causing tolerance, for MC-mediated SCD pathobiology (Vincent *et al.*, Manuscript under review). Cannabinoid receptor 1 (CB1R) mostly contributes to peripheral nociception and hyperalgesia, whereas cannabinoid receptor 2 (CB2R) is predominantly involved in MC-mediated neurogenic inflammation. Therefore, our studies with different MC stabilizers, receptor-specific MC inhibitors and analgesics demonstrate efficacy of targeting MCs to reduce neurogenic inflammation and pain in SCD. We believe that these approaches can be extended to a number of disease pathologies, which may initiate a new direction for managing pain.

### 3.12. Other MC-Involved Pain Models

In other pain-associated conditions, apart from the ones discussed above, MC-mediated nociceptive sensitization has been implicated. In a *post-operative hyperalgesia* in rats increased MC-degranulation and histamine release has been reported [239]. MC stabilizer cromolyn and ketotifen, and MC-depleting Compound 48/80 reduced post-operative pain in rats [240,241]. The MC-mediated post-operative nociceptive sensitization has been proposed to be activated by tryptase-PAR2 axis in rats [241]. Peripheral and central sensitization via tryptase-PAR2-TRPV1/TRPV4/TRPA1 pathways correlated with MC degranulation in a mouse model of paclitaxel-induced *neuropathic pain* [242]. Another rodent neuropathic pain model of spinal nerve injury demonstrated thalamic MC-mediated central sensitization in female mice, but not in male mice [36]. The pain models, discussed above, provide evidence that MCs contribute to nociception.

## 4. Future Directions

Chronic pain is a debilitating condition tremendously affecting quality of life for the patients and is a co-morbidity in a number of pain-associated disorders. Current pain treatment strategies mostly rely on use of NSAIDs, acetaminophen, opioids, anti-depressants and anti-convulsants [243]. Though these conventional treatments and other newly adopted ones (such as topical oral analgesics, triptans for migraines and cannabinoids) have been in use as primary pain medications, their efficacy generally is poor and varies between patients.

We show that MCs act, in part, as an intermediary between the disease pathology and associated pain—thus contributing to both. Therefore, MC-involved pain mechanisms need to be systematically assessed in a disease-specific manner to treat the pain symptoms with increased efficacy. Additionally, an approach to delineate the role of MC subtypes, *i.e.*, tryptase containing (MC-T type) and both tryptase/chymase-containing (MC-TC type) may be of advantage in understanding the existing mechanistic diversity. Studies implicating differential role of MC subtypes are lacking. However, there are some data on MC-subtypes in nociceptive sensitization in interstitial cystitis [77], and chronic pancreatitis and pancreatic cancer [230] . Future exploration of such subtype-specific interaction is warranted. Multi-disciplinary approaches for quantification of pain and elucidating mechanistic contribution of MCs to pain pathobiology in animal models are required to improve analgesic strategies. Inhibiting MC degranulation by MC stabilizers and/or other mast cell inhibitors may improve analgesic outcomes of opioid and/or other analgesic therapies. One such therapeutic strategy might be to use the endogenous cannabinoid-like compound *N*-palmitoyl-ethanolamine (PEA). PEA has anti-inflammatory and analgesic properties and reduces MC degranulation [244]. PEA has been shown to be effective in reducing neuropathic [245] and endometriotic pain [246,247,248] in pre-clinical and clinical studies. PEA is a endogenous molecule with minimal side effects which is known to enhance efficacy of cannabinoid receptor and TRPV1 receptor-specific treatments, referred to as entourage effect [249]. We have demonstrated that sub-optimal morphine treatment response is improved by co-treatment with MC stabilizer cromolyn in sickle mice [34]. We also showed that nociceptin agonist AT-200 ameliorates hyperalgesia in sickle mice with a concomitant decrease in mast cell activation. Therefore, a therapeutic approach using cannabinoids, nociceptin agonists, PEA and MC stabilizers in addition to conventional analgesics may lead to improved analgesia without tolerance.

## Appendix A

**Table A1:** Summary of disease-specific MC-mediated nociceptive mechanisms.

Pain Model	Proposed Role of Mast Cells	Inhibitors	References
Mast cell disease	MC being the major player in mast cell disease it is hard to pinpoint a single mechanism or pathway for pain in MCAD.	MC stabilizers, antihistamines, leukotriene inhibitors along with conventional opioids.	HS [39,40,41]
Migraine	Mediators released from dural MCs can sensitize meningeal nociceptors. Histamine can enhance nociception via histamine receptors on meningeal nociceptors. MC-mediated SP and CGRP released from trigeminal nerves can also contribute to further MC activation and continuous sensitization. Serotonin has an anti-nociceptive effect and can block MC activation at the meningeal level. MC mediators sensitize neuronal cells via acid-sensing ion channels ASIC3 (not TRPV1) in MC-induced low pH conditions at the dural level.	H1R antagonist mepyramine; Serotonin-1 receptor agonists dihydroergotamine and sumatriptan; ASIC3 blocker APETx2.	HS [47,48,49,50,51,52,53,63] RM [35,57,58,59,60,61,62,64,65,66,70] FM [63]
Pelvic pain *IC/BPS*	SP released from bladder nerve fibers activates MCs via NK1R. In turn MC-derived histamine can further sensitize the primary afferent fibers via histamine receptors, and subsequently may contribute to increased central nociception via CaMKII induction. NGF may also play an important role in MC-mediated IC/BPS.	H1R antagonists—diphenhydramine, hydroxyzine, cetirizine (10 mg/kg); H2R antagonists—cimetidine, ranitidine, famotidine; NK1R antagonists—gabapentin and L-703,606; CaMKII inhibitor KN-93.	HS [74,75,76,77,78,79,80,81,82,83,84,85,86] RM [87,88,89,90,91,95,96,97,98,99,100,250]
*CP/CPPS*	Increased MC counts and associated degranulation may induce tryptase-PAR2 activation of sensory DRG neurons while histamine may promote peripheral sensitization. CCL2 and CCL3 can also contribute to MC correlated central sensitization. NGF released from nerve endings by MC activation may contribute to further MC recruitment.	Cromolyn; H1R antagonist cetirizine; H2R antagonist ranitidine; NGF neutralizing antibody AB-256-NA.	HS [102,103,104,106,107] RM [102,105,107,108]
*CPPW*	DAMPs from menstruation can activate MCs via TLRs, and MC degranulation products can lead to TRPV1-mediated nociceptive sensitization. Sensitized neurons can release SP and CGRP leading to increased MC activation. Also, sustained activation of MCs by estrogen can be a result of enhanced biosynthesis of estrogen via prostaglandin released from MC.	Anti-CCL2/JE (AB479NA) and anti-CCL3 (AB450NA) Leukotriene antagonist zafirlukast.	HS [31,111,112,113,114,115,116,117] RM [124,125]
Irritable Bowel Syndrome	Direct nerve fiber and mast cell structural interaction may induce pain. Histamine may be involved in IBS pain as well. Tryptase-PAR2 axis via TRPV1 and Ca^2+^ mobilization can contribute to sensitization of enteric nerves. Tryptase can contribute epithelial barrier dysfunction as well. MC mediator NGF can promote visceral hyperalgesia via modulation of DRG neuron plasticity and also via mucosal dysfunction to promote MC-nerve fiber interaction. Pathologic MC activation is seen in most therapy-resistant IBS patients.	MC stabilizer cromolyn; MC stabilizer with H1R antagonist property ketotifen.	HS [32,43,134,135,136,137,138,140,141,142,145,147,148,152,153,154,160,251] RM [144,152,153,157,158,159]
Vulvodynia	Increased MC infiltration, MC degranulation and hyper-innervation in vulvar region are associated with peripheral nociceptive sensitization and hyperalgesia. Allergic MC activation may also be a contributing factor.	-----------------	HS [163,164,165] RM [166]
Complex regional pain syndrome	SP signaling mediates MC activation and correlated hypersensitivity via NK1 receptors. Increased and activated MCs may also activate keratinocytes and lead to PAR2-TRPA1 mediated nociception.	NK1R antagonist LY303870	HS [173] RM [145,174,175,176,177,179,180,181,182,183]
Venom-induced hyperalgesia	MC degranulation may excite the voltage gated sodium channels of the peripheral and central neuronal cells. MC derived histamine sensitizes the nerve fibers.	MC stabilizer cromolyn; H1R antagonists chlorpheniramine, pyrilamine; H2R antagonist cimetidine..	RM [184,185,186,187]
Fibromyalgia	Hypothalamic-pituitary-adrenal axis (HPA) may be involved in MC-mediated nociception. CRH released from HPA and also SP released from nerve fibers can activate MCs initiating a neuroendocrine cycle of feed-forward nociceptive sensitization.	MC stabilizer with histamine antagonistic property ketotifen.	HS [203,204,207]
Self-injurious behavior associated pain	Stress and abnormal innervation induced SP release may contribute to sustained activation of nearby MCs. MC mediators can further sensitize primary afferent fibers contributing to sustained pain.	MC stabilizer cromolyn.	HS [217,218,219,220]
Cancer pain	MC activation by morphine may lead to increased release of SP from nerve endings via tryptase-PAR2 axis, and SP can further contribute to sustained neurogenic inflammation and pain in cancer.	------------------	HS [226,227,228,229,230] RM [231]
Sickle cell pain	MC-derived tryptase (via PAR2-TRPV1) contributes to SP and CGRP release from peripheral nerve fibers. SP can further activate MC and MC mediators can further sensitize peripheral and central nervous system leading to sustained pain and hyperalgesia. MC activation by opioids such as morphine imparts resistance to therapy.	MC stabilizer Cromolyn; MC inhibitor Imatinib; Nociceptin agonist AT-200.	HS [234,235,236] RM [34,238]
Post-operative hyperalgesia	MC-derived tryptase may contribute to pain via PAR2.	MC stabilizer cromolyn and ketotifen; PAR2 antagonist ENMD-1068.	RM [122,239,241]
Neuropathic pain	Tryptase-PAR2-TRPV1/TRPV4/TRPA1 pathway contributes to MC-mediated pain.	PAR2 antagonist FSLLRY-amide.	RM [36,242]

## Abbreviations

MC—Mast cells; MCAD—mast cell activation disease; SP—substance P; CGRP—calcitonin gene-related peptide; TRPV1—transient receptor potential cation channel subfamily V member 1; NGF—nerve growth factor; IC/BPS—interstitial cystitis/bladder pain syndrome; CP/CPPS—chronic prostatitis associated chronic pelvic pain syndrome; CaMKII—Ca^2+^/calmodulin-dependent protein kinase II; CCL—chemokine (C–C motif) ligand; CPPW—chronic pelvic pain in women; H1R & H2R—histamine 1 and histamine 2 receptors; ASIC—acid sensing ion channels; NK1R—neurokinin 1 receptor; PAR2—proteinase activated receptor 2; HS—human studies; RM—rodent models; FM—feline models; DAMP—damage associated molecular pattern; TLR—toll like receptor; IBS—irritable bowel syndrome; TRPA1—transient receptor potential cation channel subfamily A member 1; BID—bis in die (twice a day); CRH—corticotropin-releasing hormone; and DRG—dorsal root ganglion.
